# Whitening and Impaired Glucose Utilization of Brown Adipose Tissue in a Rat Model of Type 2 Diabetes Mellitus

**DOI:** 10.1038/s41598-017-17148-w

**Published:** 2017-12-01

**Authors:** Constantin Lapa, Paula Arias-Loza, Nobuyuki Hayakawa, Hiroshi Wakabayashi, Rudolf A. Werner, Xinyu Chen, Tetsuya Shinaji, Ken Herrmann, Theo Pelzer, Takahiro Higuchi

**Affiliations:** 1 Department of Nuclear Medicine, University Hospital Würzburg, Würzburg, Germany; 2 Department of Internal Medicine I, Division of Cardiology, University Hospital Würzburg, Würzburg, Germany; 3 The Russell H Morgan Department of Radiology and Radiological Science, Division of Nuclear Medicine, Baltimore, MD United States; 4 Comprehensive Heart Failure Center, University Hospital Würzburg, Würzburg, Germany; 5 Department of Bio-Medical Imaging, National Cerebral and Cardiovascular Center, Suita, Japan

## Abstract

Brown adipose tissue (BAT) is an attractive therapeutic target to combat diabetes and obesity due to its ability to increase glucose expenditure. In a genetic rat model (ZDF fa/fa) of type-2 diabetes and obesity, we aimed to investigate glucose utilization of BAT by ^18^F-FDG PET imaging. Male Zucker diabetic fatty (ZDF) and Male Zucker lean (ZL) control rats were studied at 13 weeks. Three weeks prior to imaging, ZDF rats were randomized into a no-restriction (ZDF-ND) and a mild calorie restriction (ZDF-CR) group. Dynamic ^18^F-FDG PET using a dedicated small animal PET system was performed under hyperinsulinemic-euglycemic clamp. ^18^F-FDG PET identified intense inter-scapular BAT glucose uptake in all ZL control rats, while no focally increased ^18^F-FDG uptake was detected in all ZDF-ND rats. Mild but significant improved BAT tracer uptake was identified after calorie restriction in diabetic rats (ZDF-CR). The weight of BAT tissue and fat deposits were significantly increased in ZDF-CR and ZDF-ND rats as compared to ZL controls, while UCP-1 and mitochondrial concentrations were significantly decreased. Whitening and severely impaired insulin-stimulated glucose uptake in BAT was confirmed in a rat model of type-2 diabetes. Additionally, calorie restriction partially restored the impaired BAT glucose uptake.

## Introduction

Obesity and its associated diseases, especially diabetes type 2, constitute one of the major public health concerns in most developed societies^1^. Taking only energetics into account, treatment of obesity by reducing caloric intake and increasing energy expenditure should be simple, however most patients fail to achieve or –more important- maintain weight loss goals through diet and exercise alone^2^. Therefore, pharmacologic and/or surgical treatment options to support patients in the fight against excessive body weight are increasingly sought for. It is currently known that body weight regulation goes beyond simple energetics and that other metabolic, behavioral, neuroendocrine, and autonomic responses play important roles in maintenance of the amount of fat reserves^3^.

Since its rediscovery in adult humans in the late 2000s, brown adipose tissue (BAT) has attracted a lot of attention as a potential target to combat obesity, and stimuli for its activation have been investigated in both animal models and humans^4–14^. As part of the mechanisms of adaptive thermogenesis, BAT enables the release of energy as heat instead of storage as energy molecules by dissociation of mitochondrial substrate oxidation from ATP production, a phenomenon mediated by uncoupling protein 1 (UCP-1^15^), which is present in BAT, but not in white adipose tissue (WAT).

Most recently, experimental murine studies reported that diet-induced obesity resulted in impaired glucose tolerance, BAT functional hypoxia and subsequent structural “whitening” that eventually led to a functional shift from thermogenesis toward lipid storage^16,17^. The loss of functional BAT in obesity might be consistent with the lack of BAT response to both insulin and cold stimulation in obese adults^18^, whereas exposure to cold in young subjects does stimulate BAT activity^4^. Preserved spontaneously activated BAT was also significantly higher in patients with low fasting plasma glucose levels (<93 mg/dl) as compared to patients with glucose levels higher than 103 mg/dl^19^. However, further knowledge about the relationship between type 2 diabetes mellitus and BAT is still very limited.

In this study, we aimed to investigate BAT glucose utilization in a well-established genetic rat model of type 2 diabetes^20^ using ^18^F-FDG PET imaging under hyperinsulinemic-euglycemic clamping conditions. Rats were set under hyperinsulinemic-euglycemic conditions to stimulated BAT glucose consumption by high serum insulin concentrations during the imaging period^21–23^, furthermore the euglycemic status permitted to avoid interference of different plasma glucose concentrations between diabetic and control animals^24^.

## Methods

### Study approval

Animal studies were performed in agreement with the Guide for Care and Use of Laboratory Animals published by the US National Institutes of Health (NIH Publication No. 85-23, revised 1996) and in compliance with the German law on the protection of animals. Animal protocols were approved by the local Animal Care and Use Committee (Regierung von Unterfranken, Germany).

### Small animal ^18^F-FDG-PET/CT imaging

Ten weeks old male Zucker diabetic fatty (ZDF; n = 11) were randomized into a no-restriction diet (ZDF-ND) group (n = 6) and a mild calorie restriction (ZDF-CR) group (n = 5), male Zucker lean (ZL) rats served as controls (n = 6) (Charles River, Wilmington, USA). All animal received water *ad libitum*. Diet intervention consisted in 3 weeks of: standard food *ad libitum* to ZL lean rats (daily food intake of 20.29 ± 1.97 g); Purina 5008 (protein 23%, fat 6.5%, carbohydrates 58.5%, fiber 4% and ash 8%; as recommended by the supplier) *ad libitum* to ZDF-ND rats (daily food intake of 36.25 ± 5.8 g) and 20 g of Purina 5008 per day (the same weight of food ZL lean consumed per day) to ZDF-CR rats, respectively. At 13 weeks of age, ZDF-ND rats (average weight, 356 ± 24 g), ZDF-CR rat (average weight, 354 ± 50 g) and controls (average weight, 290 ± 25 g) underwent ^18^F-FDG-PET at room temperature (23 °C) using a dedicated small animal PET scanner (Inveon; Siemens Medical Solutions, Erlangen, Germany).

**Figure 1:**
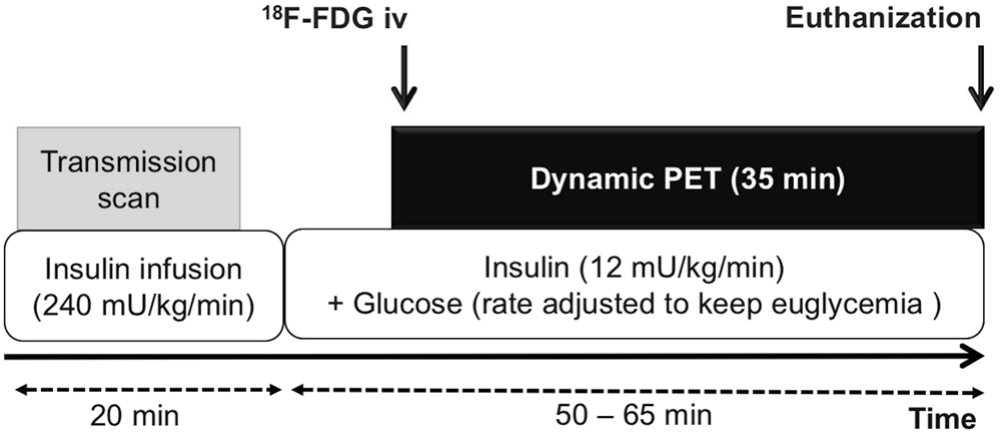
Schematic diagram illustrating the ^18^F-FDG-PET imaging protocol. Dynamic ^18^F-FDG-PET scans were performed using hyperinsulinemic-euglycemic clamping which consisted of high-dose (240 mU/kg/min) and subsequent low-dose (12 mU/kg/min) insulin plus glucose infusion (at variable rates) to obtain hyperinsulinemic-euglycemic conditions. iv = intravenous injection.

General anesthesia was induced with isoflurane after fasting (>10 h). In order to maintain euglycemia as well as to stimulate glucose consumption of BAT, hyperinsulemic-euglycemic clamping^24^ was performed in each group (Fig. 1). Two catheters were placed into the tail veins for connection to the clamping system. Infusion of insulin (Insuman Rapid; Sanofi-Aventis, Paris, France) was started at a rate of 240mU/kg/min for 20 min followed by low-dose 12 mU/kg/min. In order to maintain euglycemia (blood glucose levels: 70–110 mg/l), varying rates of glucose (50.0% glucose solution) were added by checking blood glucose levels every 2 min. ^18^F-FDG (30-45MBq) was administrated intravenously at least 12 min after stable euglycemia had been established. Dynamic emission PET acquisition (35 min) was started just before tracer administration. A thirteen-minute transmission scan with a ^57^Co rotating source was also conducted prior to the emission scan. Data was reconstructed using ordered subset expectation maximization 2D algorithm with attenuation correction. Tracer uptake in BAT as percentage injected dose (%ID) was determined by assigning free-hand volumes of interest around foci at interscapular BAT area.

### *Ex-vivo* tissue analysis

After completion of the PET scan, the animals were euthanized for *ex-vivo* BAT tissue analysis. Visual interscapular fat depots were resected and weighed.

For histological analysis, frozen specimens were sliced into 7-µm sections. Tissue sections were fixed (10-min acetone) and blocked with 10% bovine serum albumin. The tissue sections were subjected to staining using standard hematoxylin and eosin (H&E) staining protocol for histology. For immunofluorescence staining, the sections were incubated overnight for primary antibodies against UCP-1 (Abcam rabbit polyclonal, 1:500; Abcam, Cambridge, UK). Alexa Fluor 488 Donkey anti-rabbit antibody (Thermo Fisher Scientific, Waltham, MA, USA) was used as a secondary antibody. Mitochondria were stained by fluorescent staining (MitoGreen, PromoCell, concentration 200 nM; PromoCell, Heidelberg, Germany) and cellular lipids were stained by Oil red O staining (Abcam, Oil Red O kit)^25^ according to the manufacturer’s instructions. Microscopic images were obtained using a Keyence BZ-9000 microscope (Keyence Corp., Osaka, Japan). Analysis of images were performed on each sample (n = 4 rats per experimental group) at ×400 magnification with Image J software. Size of unilocular adipocytes were measured in H&E stained sections (6 fields per sample), and area of positively stained area was calculated for UCP-1 (8 per sample) and mitochondria staining (4 per sample).

Protein expression of interscapular fat depots was analyzed by Western blotting according to standard protocols (Bio-rad, Trans-Blot Turbo System, Hercules, California, USA)^26^. The following antibodies were employed: UCP-1 (Abcam rabbit polyclonal, 1:500; Abcam) and acetyl-Co A Carboxylase (Cell Signaling, rabbit polyclonal, 1:500, Cambridge, UK). Immunoreactive proteins were visualized by HRP-coupled antibodies (GE, Amersham, UK) and ECL. The ImageQuant software (Biometra, Göttingen, Germany) was used for densitometric analysis based on peak area; β-actin was employed as internal standard (Santa Cruz, mouse monoclonal, 1:200, Dallas, USA).

### Statistical analysis

All data are presented as mean ± standard deviation. Statistical analysis was performed using GraphPad Prism Software. Statistical significance between the three groups was determined by one-way ANOVA followed by post hoc Tukey multiple comparison analysis. Differences between the means were considered statistically significant if *p* < 0.05.

## Results

### Imaging results

**Figure 2:**
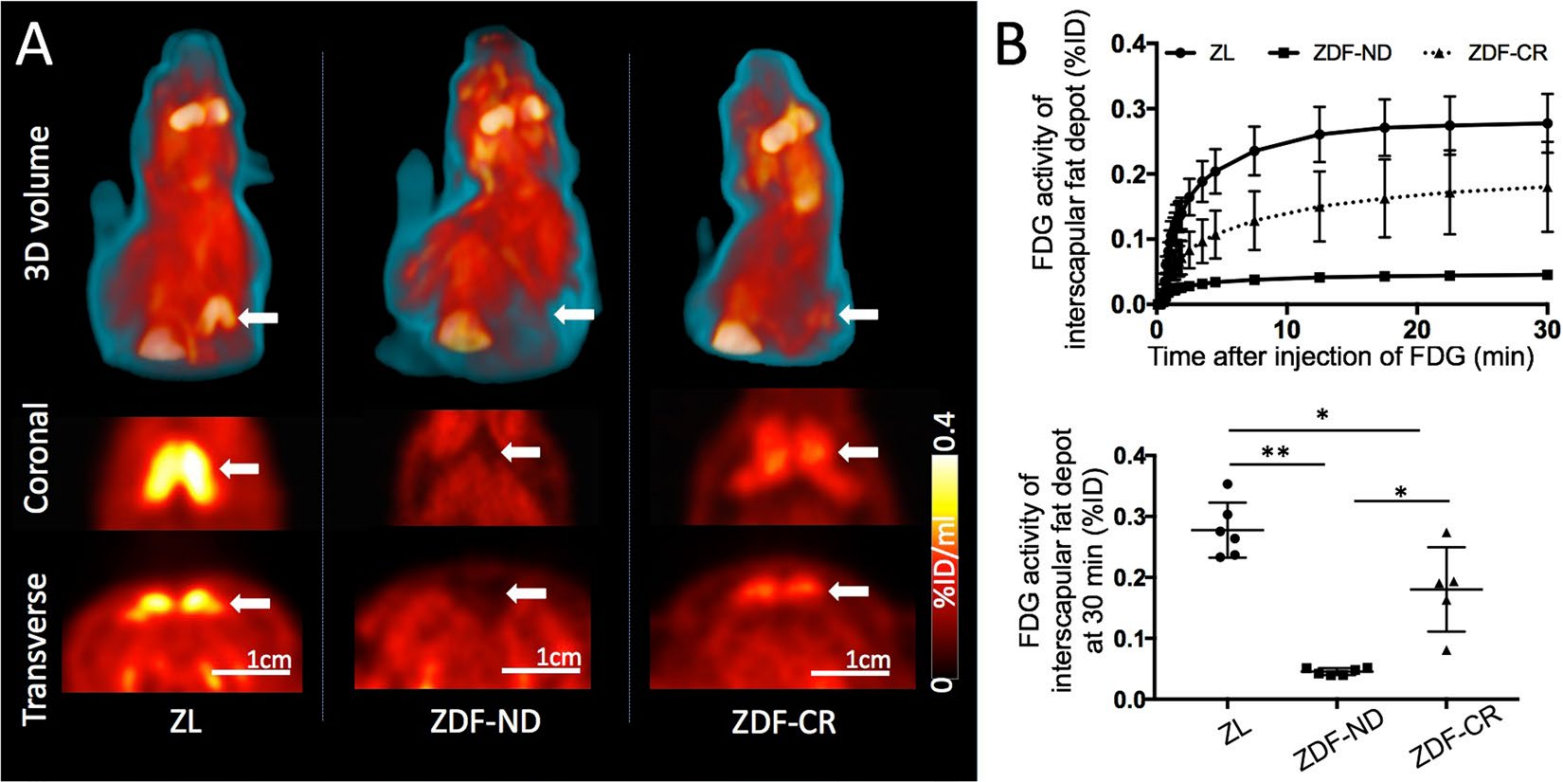
**(A)** Representative ^18^F-FDG PET images of ZL control (left), diabetic ZDF rat on a no-restriction diet (ZDF-ND, middle) and diabetic ZDF rat after three weeks of mild calorie restriction (ZDF-CR, right). Intense focal ^18^F-FDG uptake of BAT in the interscapular area was clearly depicted in the ZL control rat, while no apparent uptake was observed in the ZDF-ND rat (arrows). After calorie restriction (ZDF-CR), mild tracer uptake was identified in the interscapular area indicating improved BAT glucose utilization. **(B)** Average time-activity curves of BAT in ZL control rats, with calorie restriction (ZDF-CR), and ZDF rats with no-restriction diet (ZDF-ND) (upper). Bar graph of FDG uptake (%ID) of BAT in ZL control, ZDF-ND, and ZDF-CR rats at the time point of 30 min after ^18^F-FDG injection (lower). BAT = brown adipose tissue. *p < 0.01, **p < 0.001 and ***p < 0.0001.

In hyperinsulinemic-euglycemic state, ^18^F-FDG PET identified intense inter-scapular tracer uptake consistent with BAT glucose utilization in all ZL control rats. BAT time activity curves demonstrated a rapid increase of tracer uptake during the initial 10 min after tracer injection followed by stable tracer retention until the end of the imaging session. In ZDF-CR rats, mild but focally increased ^18^F-FDG uptake was visible in all rats. In contrast, no focally increased ^18^F-FDG uptake could be detected in ZDF-ND rats at any time point (Fig. 2).

The ^18^F-FDG uptake (%ID) of interscapular BAT at 30 min after tracer injection was calculated as 0.045 ± 0.006*,***, 0.28 ± 0.05** and 0.18 ± 0.07 in ZDF-ND rats, ZL control rats, and ZDF-CR rats, respectively, demonstrating significant differences between each group (*p < 0.01 and **p < 0.001 vs ZDF-CR, and ***p < 0.0001 vs ZDF-ND, Fig. 2B).

### Histological analysis

**Figure 3:**
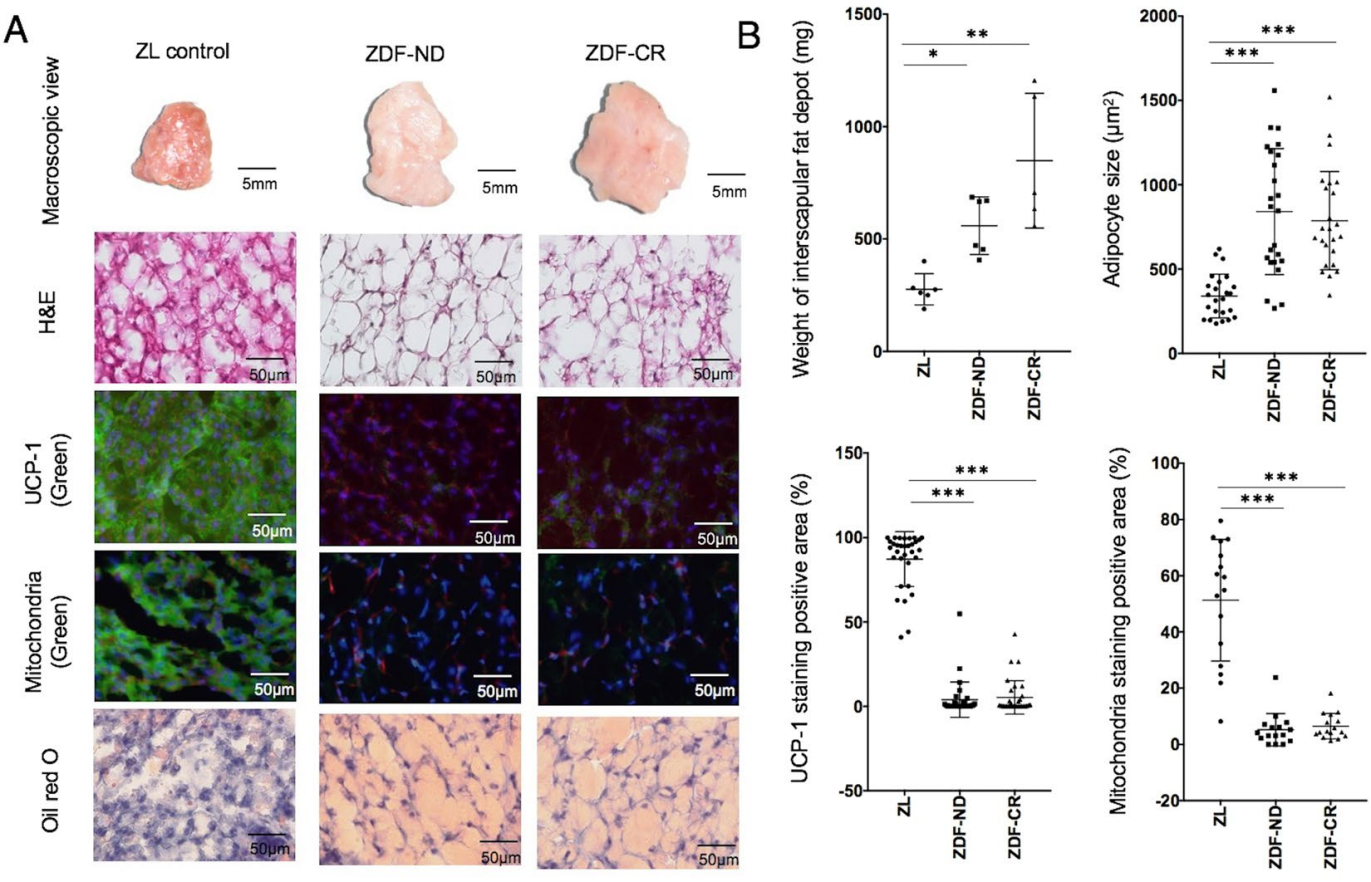
**(A)** Macroscopic, and microscopic view of H&E, immunofluorescence staining for UCP-1, fluorescence staining for mitochondria (MitoGreen), and Oil red O staining of interscapular fat depot in a ZL control, ZDF rat with no-restriction diet (ZDF-ND) and a ZDF rat with calorie restriction (ZDF-CR). In immunofluorescence staining for UCP-1 (green) and fluorescence staining for mitochondria (green) nuclei were stained with DAPI (blue) and cell membranes with wheat germ agglutinin (red). Visual analysis demonstrates a reduction in mitochondria and UCP-1 expression as well as an increase in fat cells in both ZDF-ND and ZDF-CR rats. **(B)** Bar graphs show weight of interscapular fat depot, size of adipocytes and percentage of positively stained area of UPC-1 and mitochondria staining in ZL control, ZDF-ND and ZDF-CR rats. *p < 0.05, **p < 0.01, ***p < 0.0001.

At the time of dissection, interscapular BAT with its typical reddish brown colour was easily detected by direct visual inspection in ZL control rats, whereas interscapular fat depots in ZDF-CR and ZDF-ND rats presented as whitish brown (Fig. 3).

**Figure 4:**
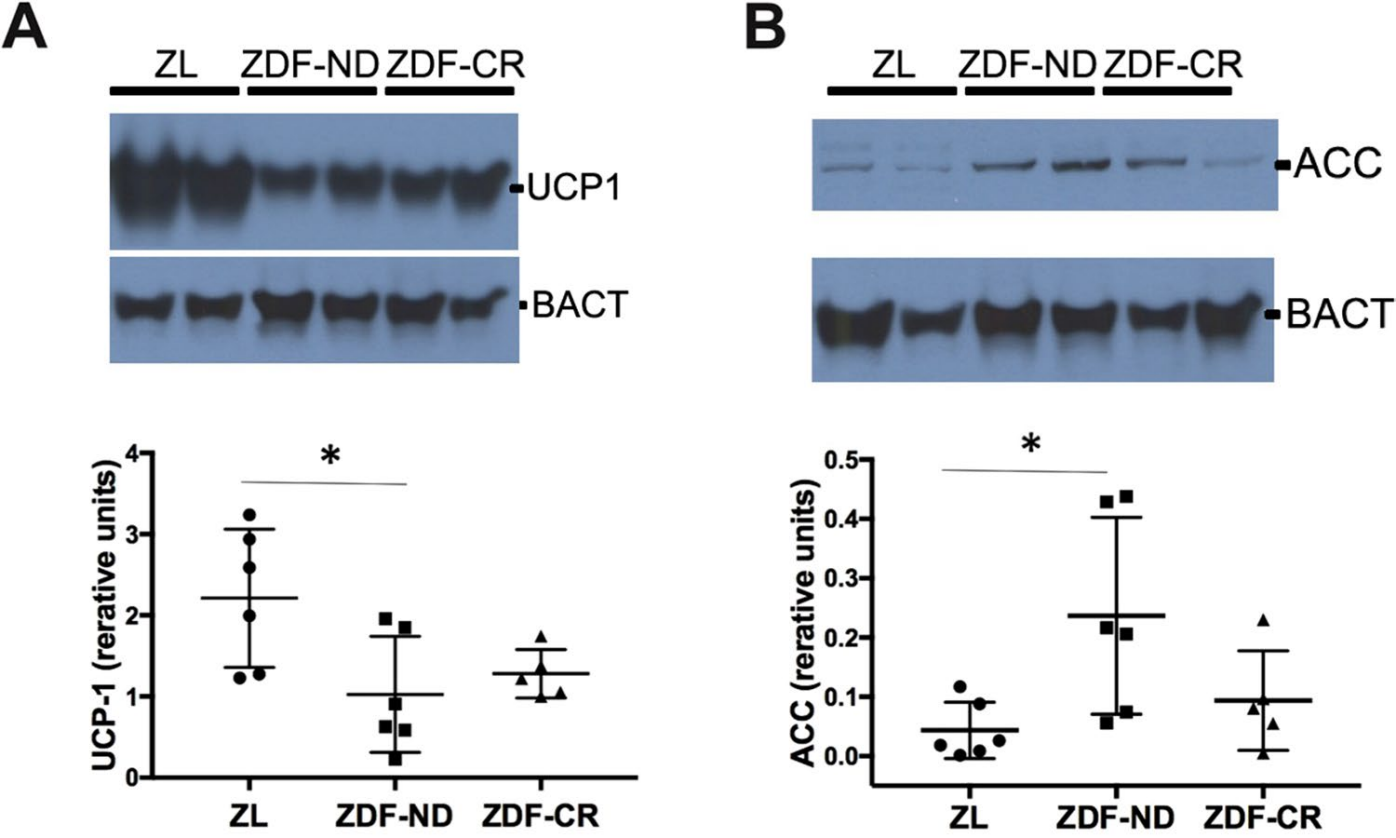
**(A)** Protein expression of the thermogenic protein UCP-1 in BAT was significantly higher in Zucker Lean (ZL, n = 6) control rats in comparison to diabetic rats with (ZDF-ND; n = 6) and without (ZDF-CR, n = 5) calorie restriction. **(B)** Protein expression of fatty acids synthesis protein acetyl-coenzyme A carboxylase (ACC) was significantly higher in BAT of ZDF-ND rats in comparison to ZL rats; ZDF-CR presented intermediate values that did not reach a significant difference to either ZL or ZDF-ND rats. β-Actin (BACT) was used as housekeeping control. *p < 0.05.

The weight of interscapular fat tissue was significantly higher in ZDF-CR and ZDF-ND rats as compared to ZL controls, and the size of adipocytes was significantly increased (Fig. 3). Microscopically, presence of brown adipocytes with abundant expression of UCP-1 in mitochondria-rich cytoplasm could be confirmed in ZL control rats. In contrast, both ZDF-ND and ZDF-CR rats presented ubiquitous white adipose-like tissue, showing large unilocular lipid droplets with decreased positive staining signals for UCP-1 and mitochondria (Fig. 3). No significant differences were identified between ZDF-ND and –CR animals. UCP-1 data was confirmed by Western blot analysis that evidenced a significantly higher expression in ZL rats in comparison to ZDF-ND and CR rats (Fig. 4).

Brown to white adipose tissue transition is accompanied by an increased synthesis and accumulation of intracellular fatty acids. The key enzyme for the biosynthesis of fatty acid is acetyl-coenzyme A carboxylase (ACC) that catalyses the irreversible carboxylation of acetyl-CoA to produce malonyl-CoA. ZDF-ND rats presented significantly increased ACC levels in BAT in comparison to ZL rats. ZDF-CR BAT presented intermediate values that were not significantly different from ZL or ZDF-ND (Fig. 4).

## Discussion

A number of previous studies have reported on impaired BAT glucose utilization in Zucker fa/fa rats^27,28^. However -to the best of our knowledge-, this is the first report directly providing PET *in vivo* data on BAT metabolism in a rat model of type 2 diabetes and obesity. In comparison to lean controls, obese diabetic ZDF rats had almost ubiquitously BAT functional loss as identified by FDG PET imaging. Morphologic transition from brown to white adipose like tissue was already evident upon visual inspection at the time of dissection and was further confirmed by histological work-up.

BAT activation has gained interest for its potential use in the treatment of obesity and diabetes type 2. Indeed, transplantation of BAT to mice has been shown to decrease body weight and improve glucose metabolism and insulin sensitivity^23^. Activation of BAT by cold exposure has been reported to lead to high consumption of glucose and lipids far exceeding hepatic and skeletal muscle capacity, therefore increased glucose uptake by BAT is considered to reflect metabolic activity and thermogenesis^3^. In fact, lower FDG uptake and whitening of BAT in the ZDF rats was accompanied by decreased protein expression levels of UCP 1 and an increase in the fatty acids synthesis enzyme ACC. Whitening of BAT seems to be involved in the process of metabolic changes in type 2 diabetes, leading to the so-called metabolic syndrome^16,29^.

In this study, all animals were analysed at an age of 13 weeks, in the mid-stage of diabetes-related morphologic and functional alterations^20^. Interestingly, calorie restriction 3 weeks prior to imaging evoked mild but significant BAT glucose utilization in ZDF rats, whereas UCP-1 expression was comparably low in ad libitum fed ZDF counterparts. This finding might be explained by calorie restriction-induced changes in glucose uptake, potentially to already improved insulin sensitivity.

Furthermore, a trend towards reduction of ACC expression after calorie restriction could be detected and indicated reduction of fatty acids synthesis in the BAT.

Unfortunately, a more profound analysis of insulin resistance as well as serial imaging of the diabetic rats at earlier and later time points to further elucidate the temporal orchestration of BAT whitening was not performed. However, insight into the beginnings of BAT whitening could be of paramount importance to assess the optimal time point for both lifestyle and dietary modifications in order to prevent complete BAT loss.

This study has several further limitations. Besides calorie restriction, we did not perform any experiments aiming at potential further interventions to re-activate uptake of small reminiscent brown or brite adipocytes or to induce browning again^30^. In a recent human study, exposure to a cold environment, a well-known BAT stimulus in healthy men^4,31^, could also be demonstrated to increase BAT glucose uptake in obese subjects^32^. Interestingly, cold acclimation did not increase glucose uptake into subcutaneous and visceral white fat depots, implying that “browning” had not occurred. Future trials should focus on therapeutic strategies to achieve browning of WAT.

Increased glucose uptake in BAT in obese subjects following cold exposure might prevent induction of the obesity-associated insulin resistance due to ameliorated glucose homeostasis. Recent studies have hinted at the importance of brown fat for glucose disposal, insulin sensitivity and triglyceride metabolism^23,33^. Though the relationship of these metabolic pathways and BAT were beyond the scope of our study, we provide new insights into the relation between type 2 diabetes and obesity, brown adipocyte alteration and metabolism.

In brief, insulin-stimulated ^18^F-FDG uptake was significantly decreased in a rat model of type 2 diabetes and correlated with transition of BAT to white adipose-like tissue. Future research needs to explore the underlying mechanistic and biological implications, as well as therapeutic options for BAT induction as, ultimately, a novel approach to BAT-targeted treatment of obesity.

## Electronic supplementary material

Supplementary Information
