# Supplementary material for: Whitening and Impaired Glucose Utilization of Brown Adipose Tissue in a Rat Model of Type 2 Diabetes Mellitus
Source: Sci Rep. 2017 Dec 1;7:16795. doi: 10.1038/s41598-017-17148-w (PMC5711946; doi:10.1038/s41598-017-17148-w)
Supplement: Supplementary file 1 — Supplementary Information [file 41598_2017_17148_MOESM1_ESM.docx]

**SUPPLEMENTARY FIGURE LEGENDS**

***Title:* Whitening and Impaired Glucose Utilization of Brown Adipose Tissue in a Rat Model of Type 2 Diabetes Mellitus**

***List of authors:*** Constantin Lapa^1^, Paula Arias-Loza^2^, Nobuyuki Hayakawa^1^, Hiroshi Wakabayashi^1^, Rudolf A Werner^1,3^, Xiynyu Chen^1,4^, Tetsuya Shinaji^1^, Ken Herrman^1^, Theo Pelzer^2*^, and Takahiro Higuchi^1,4,5*^

*^1^Department of Nuclear Medicine, University Hospital Würzburg, Würzburg, Germany*

*^2^Department of Internal Medicine I, Division of Cardiology, University Hospital Würzburg, Würzburg, Germany*

*^3^The Russell H Morgan Department of Radiology and Radiological Science, Division of Nuclear Medicine, Johns Hopkins University School of Medicine, Baltimore, MD, United States*

*^4^Comprehensive Heart Failure Center, University Hospital Würzburg, Würzburg, Germany*

*^5^Department of Bio-medical Imaging, National Cerebral and Cardiovascular Center, Suita, Japan.*

**SUPPLEMENTARY FIGURE LEGENDS**


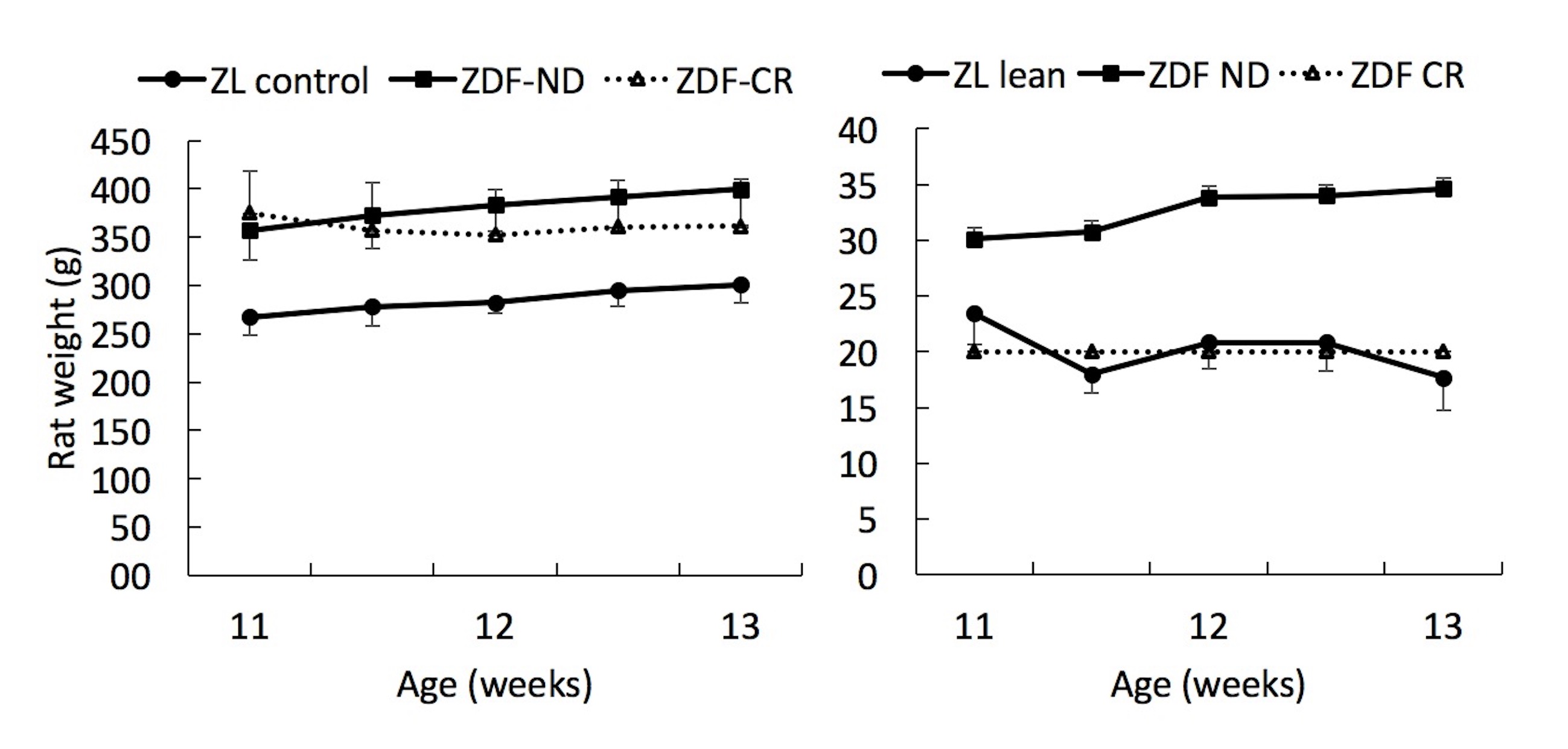


**Supplementary Figure 1:** Display of both body weight and daily food intake for all three animal groups over time, respectively.

**Full Blots for Figure 4**


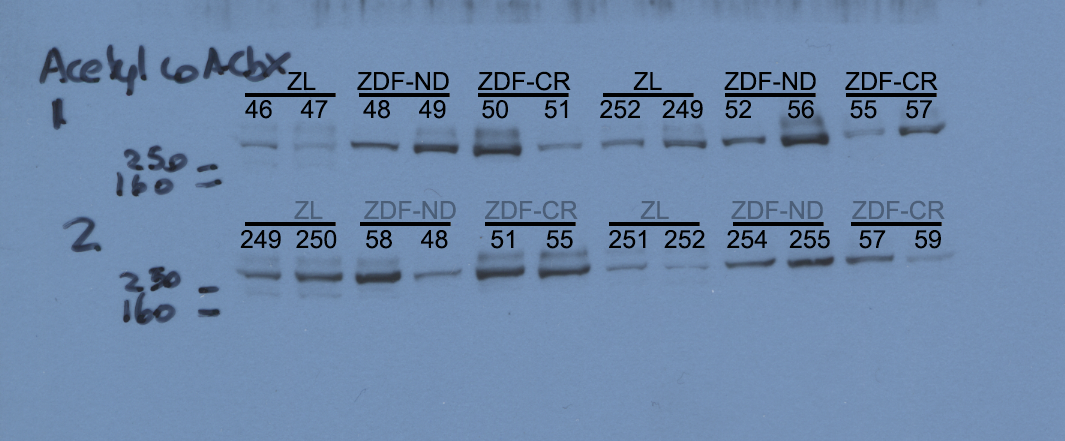


**2**

**1**

**Supplementary Figure 2.** Original Full Blots of protein acetyl-coenzyme A carboxylase (ACC) in Figure 4. Western blotting was performed according to standard protocols (Biorad Turboblot System). Immunoreactive proteins were visualized by HRP-coupled antibodies (Amersham) and ECL. The signal was detected in films developed in an ECOMAX PROTEC (Medical Systems) developing machine. The films were scanned in the ImageQuant software (Biometra) and were used for densitometric analysis based on peak area. 1 and 2 mark two independent gels. The red box marks the part of the Blot used for figure 4.


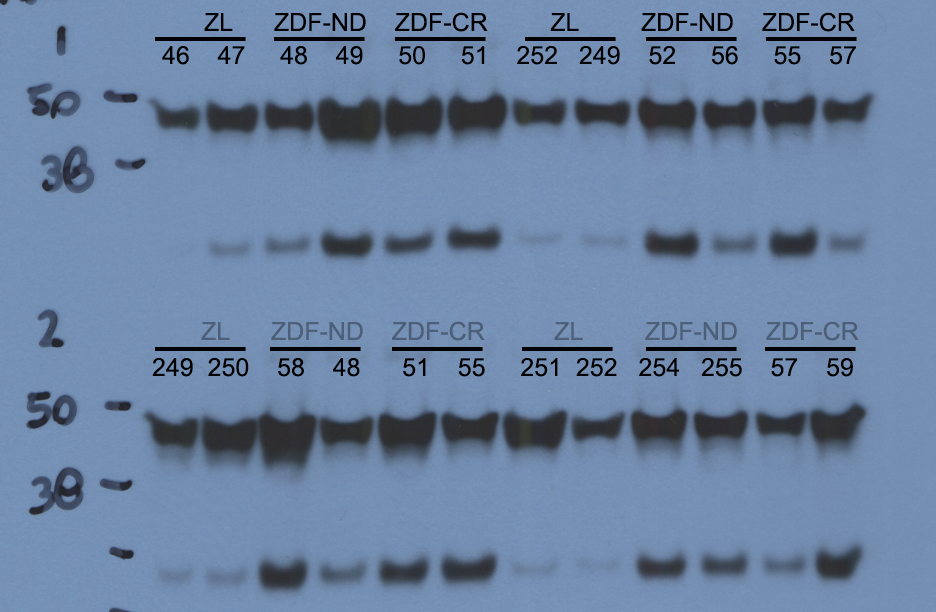


**2**

**1**

**Supplementary Figure 3.** Original Full Blots of protein β-Actin (BACT), corresponding to the acetyl-coenzyme A carboxylase (ACC) gel in Figure 4. Western blotting was performed according to standard protocols (Biorad Turboblot System). Immunoreactive proteins were visualized by HRP-coupled antibodies (Amersham) and ECL. The signal was detected in films developed in an ECOMAX PROTEC (Medical Systems) developing machine. The films were scanned in the ImageQuant software (Biometra) and were used for densitometric analysis based on peak area. 1 and 2 mark two independent gels. The red box marks the part of the Blot used for figure 4.


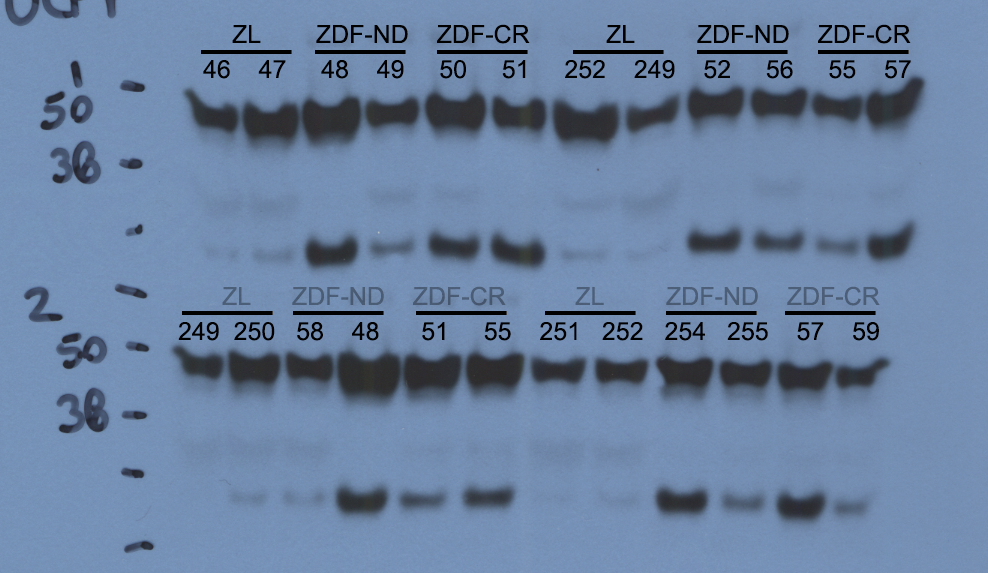


**2**

**1**

**Supplementary Figure 4.** Original Full Blots of protein β-Actin (BACT) corresponding to UCP1, in Figure 4. Western blotting was performed according to standard protocols (Biorad Turboblot System). Immunoreactive proteins were visualized by HRP-coupled antibodies (Amersham) and ECL. The signal was detected in films developed in a ECOMAX PROTEC (Medical Systems) developing machine. The films were scanned in the ImageQuant software (Biometra) and were used for densitometric analysis based on peak area. 1 and 2 mark two independent gels. The red box marks the part of the Blot used for figure 4.


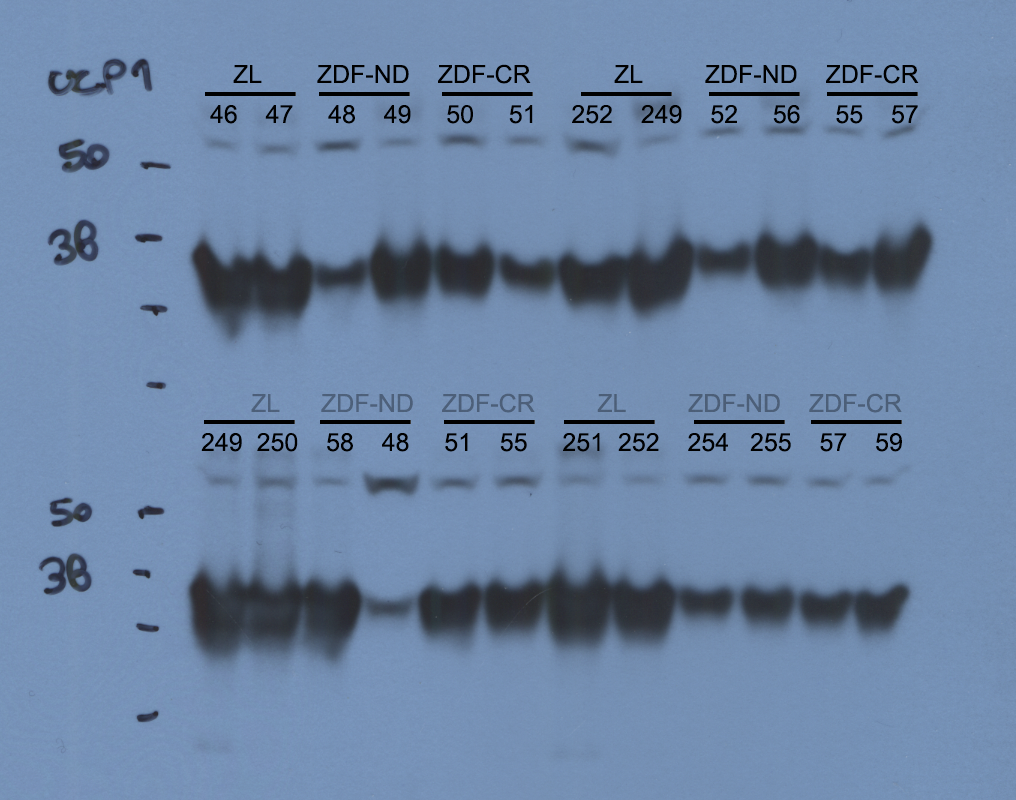


**2**

**1**

**Supplementary Figure 5.** Original Full Blots of protein UCP1, in Figure 4. Western blotting was performed according to standard protocols (Biorad Turboblot System). Immunoreactive proteins were visualized by HRP-coupled antibodies (Amersham) and ECL. The signal was detected in films developed in a ECOMAX PROTEC (Medical Systems) developing machine. The films were scanned in the ImageQuant software (Biometra) and were used for densitometric analysis based on peak area. 1 and 2 mark two independent gels. The red box marks the part of the Blot used for figure 4.
